# The Role of Polyphenols, ***β***-Carotene, and Lycopene in the Antioxidative Action of the Extracts of Dried, Edible Mushrooms

**DOI:** 10.1155/2010/173274

**Published:** 2010-12-23

**Authors:** A. Robaszkiewicz, G. Bartosz, M. Ławrynowicz, M. Soszyński

**Affiliations:** ^1^Department of Molecular Biophysics, University of Łódź, Banacha 12/16, 90-237 Łódź, Poland; ^2^Department of Biochemistry and Cell Biology, University of Rzeszów, St. Pigonia 6, 35-959 Rzeszów, Poland; ^3^Department of Mycology, University of Łódź, Banacha 12/16, 90-237 Łódź, Poland

## Abstract

One of the nutritional benefits of mushrooms is the presence of bioactive secondary metabolites which have been reported to exert various beneficial effects *in vivo*. Therefore, we selected thirteen frequently consumed species of Polish mushrooms and determined the concentration of polyphenols, flavonoids, *β*-carotene, and lycopene in aqueous and methanolic extracts of dried fruiting bodies as well as their reducing power and ability to scavenge ABTS cation radical. We found that the concentration of antioxidants is different in different species and in various parts of the fruiting body of mushrooms. We observed a strong correlation (*r* > 0.9) between the concentration of total phenolics and reducing power/scavenging effects in both aqueous and methanolic extracts, while this correlation was moderate for flavonoids. Beta-carotene did not contribute discernibly to the antioxidative properties of the extracts, while lycopene had a significant contribution to the scavenging activity of methanolic mushroom extracts.

## 1. Introduction

Mushrooms have been exploited in human diet for centuries because of their specific taste and flavour. Nowadays, they attract attention because of their beneficial effects and possible use in the prevention or treatment of diseases [1]. Numerous reports demonstrate beneficial *in vivo* effects of cultivated and wild edible mushrooms. It has been proven that the polysaccharide extract of *Pleurotus pulmonarius* delays the progression of hepatocellular carcinoma [2]; polysaccharide from *Pholiota nameko* has anti-inflammatory properties in rodents [3]; *Agaricus bisporus* inhibits prostate tumor growth in mice [4]; *Pleurotus eryngii, Grifola frondosa*, and *Hypsizygus marmoreus* protect apolipoprotein-E deficient mice from development of atherosclerosis [5]. Simultaneously, edible mushrooms are regarded as an important dietary supplement for people interested in calorie restriction, because of the low amount of fat, cholesterol, and calories in their bodies and high concentration of fiber [1, 6–8]. 

The therapeutic action of mushrooms is attributed to the presence of bioactive compounds such as vitamins, polysaccharides, and secondary metabolites in their fruiting bodies. Some of them have antioxidant properties which are referred repeatedly to be the key aspect of their observed beneficial effects. Polyphenols and carotenoids, abundant in the fruiting bodies of mushrooms, are antioxidants efficient in biological systems [9]. Polyphenols have been reported to interfere with the initiation and progression of cancer [10, 11], to act as antiageing [12], anti-inflammatory [13, 14], and brain-protective factors [15] and to protect against cardiovascular diseases [16, 17]. Apart from provitamin A properties, carotenoids are known as singlet oxygen quenchers [18, 19] and lipid peroxidation chain breakers [20]. They have been reported to reduce the risk of prostate cancer [21, 22], digestive tract cancers [23, 24], and chronic diseases [25–27].

Basing on this knowledge, we decided to estimate parameters describing the antioxidant properties of extracts of dried, edible Polish mushrooms: total antioxidant capacity with two most commonly used assays (Trolox equivalent antioxidant capacity and ferricyanide reducing power) and the content of polyphenols and flavonoids, the main compounds contributing to the antioxidant properties of mushrooms as well as of two carotenoids (*β*-carotene and lycopene), important from the point of view of antioxidant protection of the human organism. We selected thirteen species frequently consumed in Poland. For some species, we distinguished subtypes, considering the Polish nutritional customs (*Boletus edulis* white-young and yellow-adult, *Agaricus bisporus* with and without peel).

## 2. Materials and Methods

### 2.1. Materials

Sodium hydroxide, hexane, acetone, and the Folin-Ciolcalteu reagent were purchased from POCh (Gliwice, Poland). All other reagents were from Sigma (Poznań, Poland).

The fruiting bodies of Polish mushrooms were collected in 2008 in Bory Tucholskie (a big forest area in the Northern part of Poland), cleaned and air dried. *Tuber mesentericum* was collected in calcareous area of Kraków-Częstochowa (Poland). Latin names of fungi are after Wojewoda [28] and Ławrynowicz [29].

### 2.2. Mushroom Extracts

Extraction was performed according to standard, commonly used procedures [30]. Before extraction, the dry fruiting bodies were cleaned carefully to remove residual contamination. 1 g of dry caps and stalks was ground into powder, then 15 ml of boiling water or methanol was added, and the material was rubbed for the next 5 min and stirred at room temperature for 15 min. Then, the mixture was centrifuged at 3000 × g at room temperature for 20 min. The supernatants were portioned and kept frozen at −23°C until analysis. The assays used to evaluate antioxidant properties of the mushrooms are typical for such type studies to enable comparisons with literature data.

### 2.3. Assay for Total Phenolics

The concentration of total phenolics in aqueous and methanolic extracts was estimated with the Folin-Ciocalteu reagent, a method most commonly used [31, 32]. The calibration curve was prepared with gallic acid (0–0.75 mg/ml).

### 2.4. Assay for Total Flavonoids

The measurement of the content of flavonoids was conducted according to the simple and reliable method described by Jia et al. [33]. 50 *μ*l of methanolic or aqueous extract was mixed with 700 *μ*l of deionized water and 37 *μ*l of 5% NaNO_2_. After 5-min incubation at room temperature, 75 *μ*l of 10% AlCl_3_ was added followed by 250 *μ*l of 1 M NaOH after the next 6 min. After shaking, the mixture was centrifuged (5000 × g, room temperature, 15 min), and the absorbance of the supernatant was read at 515 nm against a blank. Quercetin (0–0.4 mg/ml) was used as a standard.

### 2.5. Assay for *β*-Carotene and Lycopene

The concentration of *β*-carotene and lycopene in mushroom extracts was estimated spectrophotometrically [34, 35]. This simple and rapid method makes use of specific spectral properties of the carotenoids. The content of *β*-carotene and lycopene was calculated from the equations given as follows:

(1) $$\begin{array}{cc} & \text{Lycopene}\left(\text{mg}/100\;\text{ml}\right)\\ & \;=-0.0458\;{\text{A}}_{663}+0.372\;{\text{A}}_{505}-0.0806\;{\text{A}}_{453},\\ & \beta \text{-Carotene}\left(\text{mg}/100\;\text{ml}\right)\\ & \;=0.216\;{\text{A}}_{663}-0.304\;{\text{A}}_{505}+0.452{\text{A}}_{453}\;. \end{array}$$

### 2.6. Trolox Equivalent Antioxidative Activity (TEAC) Measurement

TEAC of mushroom extracts was estimated by a modified [36] ABTS cation radical decolorization assay [37]. This simple and cheap assay is based on the measurement of the extent of reduction of preformed ABTS radical cation by antioxidants [36]. The modification used by us consists in measurement of the reduction at the wavelength of 414 nm corresponding to the main absorption peak of ABTS radical cation to increase the sensitivity of the assay [35]. ABTS radical cation was prepared according to Re et al. by reaction of ABTS with potassium persulfate and stored at −20°C until use. The standard curve was prepared using Trolox as a standard (0–20 *μ*M).

### 2.7. Reducing Power

The ability of the extracts to reduce potassium ferricyanide was determined according to Puttaraju and coauthors. This assay was reported to be more selective for antioxidants than the ABTS decolorization assay and has been used by previous researchers to study mushrooms [30]. We increased the volumes of reagents to obtain the final volume of the reaction mixture readable in a standard spectrophotometer (about 1 ml). The method was used to enable comparison with results of other studies employing this assay. 25 *μ*l of mushroom extracts was mixed with 475 *μ*l of sodium phosphate buffer (200 mM, pH 6.6) and 250 *μ*l of 1% potassium ferricyanide and incubated at 50°C for 20 min. Then, 250 *μ*l of 10% TCA was added; the mixture was shaken and centrifuged (5000 × g, room temperature, 10 min). Subsequently, 500 *μ*l of the supernatant was mixed with 500 *μ*l of deionized water and 100 *μ*l of 0.1% ferric chloride. After vigorous shaking and 10 min incubation at room temperature, the absorbance was measured at 700 nm against a blank. For the standard curve, gallic acid was employed (0–0.5 mg/ml).

### 2.8. Statistical Analysis

The dependence of TEAC/reducing power on the concentrations of individual antioxidants analyzed is presented as Pearson's correlation coefficients, and the statistical significance of the correlation coefficients was tested with Student's *t*-test. The regression was calculated by the least-squares method.

## 3. Results

### 3.1. Concentration of Total Phenolics

The content of total phenolics differed significantly among the species employed for the experiments and ranged from 1.65 ± 0.10 to 13.01 ± 1.48 *μ*g/mg of dried mushrooms in the aqueous extracts and from 0.02 ± 0.02 to 4.85 ± 0.30 *μ*g/mg in the methanolic extracts of dried mushrooms (Table 1). In general, the concentration of total phenolics was higher in aqueous than in methanolic extracts, except for the stalk of *Suillus bovinus*. We found that the content of total phenolics was different in different parts of the fruiting bodies. In eight out of nine pairs of mushroom caps and stalks analyzed, the concentration of water-soluble phenolics was higher in the caps. The content of methanolic-soluble phenolics was higher the in caps in six pairs and in the stalks in three pairs analyzed. The highest concentration of methanolic- and water-soluble phenolics was found in the caps of white *Boletus edulis* and *Xerocomus subtomentosus* and the lowest in *Tuber mesentericum*.

### 3.2. Concentration of Flavonoids

The highest total content of flavonoids (in aqueous and methanolic extracts) was found in *Xerocomus badius* and *Leccinum* spp., while the lowest in *Tuber mesentericum* and *Cantharellus cibarius* (Table 1). Similarly to the experiment with polyphenols, more flavonoids were extracted with water except for a few species, for which the concentration of flavonoids was higher in the methanolic extracts or similar in aqueous and in methanolic extracts. Like in the case of total phenolics, we found differences between the content of flavonoids in caps and stalks, and it should be emphasized that, in eight out of nine species, their concentration was higher in the caps. The Pearson correlation coefficient between the content of total phenolics and flavonoids indicates a moderate dependence: *r* = 0.69 ± 0.11 (*P* ≤ .001) and *r* = 0.59 ± 0.14 (*P* ≤ .01) for aqueous and methanolic extracts, respectively.

### 3.3. Concentration of *β*-Carotene and Lycopene

The content of *β*-carotene differed considerably between the analyzed edible mushroom species, from 0.233 to 15.256 *μ*g/g of dried body (Table 2). The highest content was found in methanolic extracts of the cap of *Tricholoma equestre* and in three species of *Suillus*. The relatively high content of *β*-carotene was detected in the aqueous extracts of the cap of *Tricholoma equestre* and *Suillus bovinus*, while *Tuber mesentericum* and the stalk of *Leccinum* spp. were deprived of this antioxidant.

The content of lycopene was far lower than the concentration of *β*-carotene in the mushrooms studied (Table 2). The highest content of lycopene was detected in the methanolic extracts of three species of *Suillus, S. bovinus* being the richest in this compound. A high content of lycopene was found in the aqueous extracts of the cap of *Suillus bovinus* as well.

### 3.4. Trolox Equivalent Antioxidative Activity (TEAC)

The ability to scavenge the ABTS cation radical ranged from 3.81 ± 0.17 to 62.30 ± 1.77 and from 0.42 ± 0.48 to 20.54 ± 1.89 Trolox equivalents/g dried mushrooms for aqueous and methanolic extracts, resp. (Table 3). In general, we observed higher TEAC values for aqueous extracts (except for *Suillus bovinus*) and differences between the analyzed parts of the fruiting bodies. Our results indicate that, for the majority of species analyzed, the aqueous and methanolic extracts obtained from caps are more potent in the decolorization of ABTS^•+^. Values of the Pearson correlation coefficient point to the strong dependence between TEAC and the total concentration of phenolics (0.95 ± 0.02 and 0.98 ± 0.01 for aqueous and methanolic extracts, resp.). A statistically significant correlation was found between the concentration of flavonoids and TEAC as well as between the concentration of lycopene and TEAC of methanolic extracts, while *β*-carotene concentration did not correlate with TEAC significantly.

### 3.5. Reducing Power

**Figure 1:**
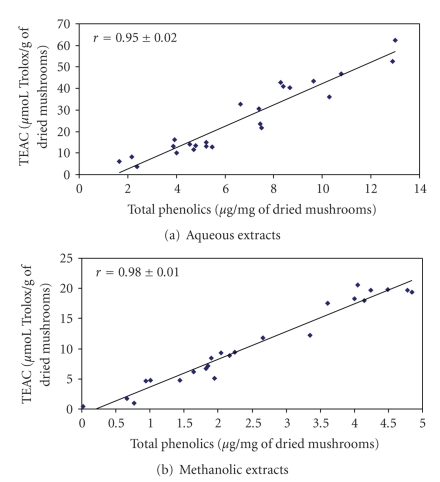
The correlation between the concentration of phenolic compounds and Trolox equivalent antioxidant capacity. The Pearson correlation coefficient between Trolox equivalent antioxidant capacity and total concentration of phenolics was calculated and tested with Student's *t*-test. The linear regression was determined with the least-squares method.

As in previous experiments, our results demonstrate differences in the ability to reduce ferricyanide between the aqueous and methanolic extracts of selected edible mushroom species as well as between the parts of their fruiting bodies (Table 3). Generally, the aqueous extracts were more potent in the reduction of ferricyanide (except for the stalk of *Suillus bovinus*). However, the relation between the reducing power of the caps and the stalks was not consistent and depended on the species analyzed. Simultaneously, we found a high, statistically significant correlation between the values of the reducing power and the concentration of total phenolics (*r* = 0.93 ± 0.03 for methanolic and 0.92 ± 0.03 for aqueous extracts) (Table 4, Figures 1(a) and 1(b)). A moderate correlation was also observed between the reducing power and the concentration of flavonoids (0.57 ± 0.14 for aqueous extracts and 0.45 ± 0.17 for methanolic extracts), while *β*-carotene and lycopene did not affect this parameter significantly.

## 4. Discussion

The beneficial effects of mushroom consumption are widely described. Studies of bioactivity of mushroom extracts suggest that the free radical scavenging ability is, at least partially, responsible for their positive action. Many papers describe the antioxidative properties and the content of antioxidants in extracts of fresh, dried, and cooked edible medicinal, cultivated, and wild mushroom species [30, 38–41]. In this study, using two *in vitro* assays, we evaluated the antioxidative ability of the extracts of 13 dried, edible mushroom species, which are especially popular in the Polish diet. We found large differences between species and between the parts of their fruiting bodies. The highest ABTS cation radical and ferrocyanide reduction ability were determined for *Boletus edulis* and *Xerocomus subtomentosus*. Many of *in vivo* studies confirm that diet supplementation with some mushroom species or mushroom extracts protects tissues against oxidative injuries [42–45]. Thus, the antioxidative ability determined in the *in vitro* experiments may be of relevance in *in vivo* systems as well. 

The antioxidative properties of mushroom extracts are dependent on the concentration of various antioxidants with different correlation coefficients. In our study, we found that both the TEAC values and the extract abilities to reduce ferricyanide showed the strongest correlation with the content of total phenolics in the dried fruiting bodies. Similar observations for mushroom species were made by other authors as well [38, 39]. Moreover, the relation between the antioxidative capacity and the concentration of polyphenols was found also for plant extracts. We found a moderate correlation between the TEAC/reducing power and the level of mushroom flavonoids. It needs to be emphasized that the dependence between the concentration of total phenolics and flavonoids is similar to that between TEAC and the flavonoids content (*r* of 0.69 ± 0.11 versus 0.68 ± 0.11 and 0.59 ± 0.14 versus 0.58 ± 0.14 for aqueous and methanolic extracts, resp.). We did not find any significant correlation between the *β*-carotene content and TAC or reducing power. Only lycopene affected the antioxidative capacity of methanolic extracts significantly, but the correlation coefficient was relatively low (0.41 ± 0.17). 

When comparing the methods of antioxidative capacity analysis employed in this study, it needs to be remarked that in the case of polyphenols, flavonoids, and lycopene, higher correlation was found between their concentrations and TEAC than reducing power values. These results suggest that both aqueous and methanolic mushroom extracts are more potent in the reduction of ABTS cation radical than in the reduction of ferricyanide. However, it should be considered that the analysis of reducing power is conducted by a more complicated procedure involving 20-min incubation at 50°C at pH = 6.6, which may affect the antioxidative capacity of the extracts. Nevertheless, the correlation between these two methods of estimation of reducing capacity remains relatively high (*r* values of 0.92 ± 0.03 and 0.89 ± 0.04 for aqueous and methanolic extracts, resp.). 

Our experiments indicate that edible Polish mushroom species differ in the content of the analyzed antioxidants. The differences were found between the parts of their fruiting bodies and between the aqueous and methanolic extracts as well. Generally, the concentration of total phenolics was much higher in aqueous than in methanolic extracts. These results are consistent with previous reports [30]. In the prevailing number of mushroom species, a similar dependence was observed for flavonoids. The values of total phenolics concentration in aqueous extracts of dried *Boletus edulis*, *Macrolepiota procera*, and *Cantharellus cibarius* obtained in our laboratory are similar to the values reported by Puttaraju and co-authors for these species collected in India [30]. For *Boletus edulis*, we found the average concentration of polyphenols to be 9.87 mg/g dried mushroom, while Puttaraju et al. reported a value of 10.2 mg/g; for *Macrolepiotea procera*, the values were 10.3 and 10.2 mg/g, respectively, and for *Cantharellus cibarius* 2.4 and 2.0 mg/g of dried mushrooms, respectively. However, in the case of the methanolic-soluble polyphenols more significant differences were observed. 

The data of Kähkönen and co-authors indicate that total concentration of phenolics in vegetables used in their experiments ranges for vegetables from ~0.4 (pea) to ~7.4 *μ*g of gallic acid equivalents per mg dried mass (leaves of carrot). For berries and fruits, the values varied from ~11.9 *μ*g/mg (apple) to ~50.8 *μ*g/mg (crowberry) while for herbs and medicinal plants from 0.2 *μ*g/mg (wheat, grain) to ~42.1 *μ*g/mg (purple loosestrife) [46]. Thus, dried mushroom bodies may be regarded as an alternative source of phenolics. Simultaneously, some mushroom species (*Suillus*) were found to be a competitive source of hydrophobic antioxidants as well. The highest concentration of *β*-carotene (~15.26 *μ*g/g of dried mushroom body) and lycopene (~15.4 *μ*g/g) were determined in the cap of *Suillus bovinus*. For comparison, the content of carotenoids reported by Ben-Amotz and Fishler in vegetables ranges from undetectable levels to ~52.8 *μ*g/g (persimmon) and ~532.1 *μ*g/g (pitango) in fruits and to ~1030 *μ*g/g (carrot) and ~243.1 *μ*g/g (tomato) for *β*-carotene and lycopene, respectively [47]. Moreover, prior studies showed that phenolics and carotenoids at the concentrations found in fruits and vegetables act as antioxidants *in vitro* and affect the antioxidant defense in human body as well. Supplementation of the daily diet with *β*-carotene (90 mg) increased the antioxidant capacity of plasma in older woman [48]; enrichment of the daily diet of nonsmoking men with tomato juice (40 mg lycopene), carrot juice (22.3 mg *β*-carotene and 15.7 mg *α*-carotene), and spicyspinach powder (11.3 mg lutein) reduced the level of oxidatively modified DNA bases in lymphocytes [49]. 30 min after consumption of 150 ml fruit juices (orange, melon, grape, peach, plum, apple, and kiwi), generation of reactive oxygen species in the plasma of healthy men was suppressed [50]. Similarly, the exercise-induced oxidative damage of red blood cells in athletes receiving 150 ml of chokeberry juice daily (34.5 mg anthocyanins) was lower when compared to control group (placebo) [51]. 

It has been documented that the chemical composition, antioxidant profile, and the concentrations of antioxidants in mushroom fruiting bodies depend on the maturation process [52, 53]. The analysis of the changes of the fundamental mushroom antioxidants in the methanolic extracts of *Lactarius piperatus* in four maturity stages demonstrated that their level increases up to the second stage of maturation and then drops down dramatically [54]. In our study, the investigation of the antioxidative capacity of immature and mature forms of *Boletus edulis* suggests that the level of water- and methanol-soluble antioxidants may be different for various parts of the fruiting body during maturation as well. The higher concentration of water-soluble polyphenols was found in the immature form, while the stalks of mature stage of this mushroom were richer in methanol-soluble polyphenols in comparison to the immature form. Thus, the aqueous extracts obtained from immature *Boletus edulis *revealed a higher ability to reduce ABTS cation radical and ferricyanide, while in methanolic extracts a higher antioxidative potential was found for the stalk. Simultaneously, a higher concentration of *β*-carotene was determined in the methanolic extract of both the caps and the stalks of the mature form. The level of lycopene was relatively low in all analyzed samples.

In order to take into account the Polish nutritional practice, we investigated the fruiting bodies of *Agaricus bisporus* with and without the peel. The results obtained for many fruits and vegetables indicate that their peel is characterized by high concentration of antioxidants and displays a protective action against oxidative agents in chemical and biological systems [55–57]. Our experiments proved that extracts of dried *Agaricus bisporus* deprived of peel are slightly less effective in scavenging ABTS cation radical and reduction of ferricyanide. Simultaneously, slightly higher levels of total phenolics and flavonoids were found in the extracts of whole constituents of the fruiting body. It cannot be excluded that the higher content of antioxidants and higher antioxidative capacity of the peel of *Agaricus bisporus* may play a physiological and, probably, protective function. Certainly, the composition of antioxidants and their role is noteworthy, and further experiments including other mushroom species need to be conducted. 

It needs to be emphasised that commercial mushroom species employed in this study (*Agaricus bisporus, Pleurotus ostreatus*) reveal relatively weak antioxidative capacity and low total content of phenolics. Likewise, it was reported previously that wild species are less energetic but possess higher concentration of polyphenols [58, 59].

However, in contrast to these optimistic results, it needs to be mentioned that even edible mushroom species have been described to demonstrate toxic effects as well. Consumption of *Tricholoma equestre* led to increase of serum creatine kinase, fatigue, and muscle weekness [60], suggesting the presence of toxin in this mushroom body which may cause rhabdomyolysis. Recently, *Agaricus bisporus* has been found to increase plasma bilirubin concentration, *Lentinus edodes* to increase plasma creatine kinase activity, and *Pleurotus ostreatus* to increase water intake and plasma alanine aminotransferase activity in mice [61]. Thus, the potential beneficial action of edible mushroom may be complicated by the possible disadvantageous effects.

## 5. Conclusions

Our results demonstrate that differences in the antioxidant profile and antioxidative capacity occur not only between dried mushroom species but also between different parts of the fruiting bodies. Moreover, we found that polyphenols rather than other antioxidants analysed affect the antioxidative ability of dried mushroom extracts.

## Figures and Tables

**Table 1:** The content of total phenolics (*μ*g of gallic acid equivalents/mg of dried mushrooms) and flavonoids (*μ*g of quercetin equivalents/mg of dried mushrooms) of edible Polish mushrooms.

Mushroom	(Total phenolics *μ*g of gallic acid equivalents/mg of dried mushrooms)		(Flavonoids *μ*g of quercetin equivalents/mg of dried mushrooms)	
	Methanolic extract	Aqueous extract	Methanolic extract	Aqueous extract
*Boletus edulis* white cap	4.49 ± 0.16	13.01 ± 1.48	0.73 ± 0.07	3.86 ± 0.10
*Boletus edulis* white stalk	3.61 ± 0.10	10.78 ± 0.33	0.52 ± 0.03	1.97 ± 0.04
*Boletus edulis* yellow cap	4.24 ± 0.13	8.28 ± 0.46	1.34 ± 0.07	1.23 ± 0.01
*Boletus edulis* yellow stalk	4.85 ± 0.30	7.39 ± 1.67	0.57 ± 0.04	3.03 ± 0.06
*Xerocomus badius* cap	1.82 ± 0.09	8.39 ± 0.65	1.81 ± 0.08	7.74 ± 0.23
*Xerocomus badius* stalk	2.66 ± 0.16	8.67 ± 0.35	1.23 ± 0.10	3.89 ± 0.02
*Leccinum* spp. cap	2.25 ± 0.14	9.63 ± 0.75	1.48 ± 0.03	6.70 ± 0.11
*Leccinum* spp. stalk	0.94 ± 0.08	3.93 ± 0.09	0.14 ± 0.01	1.74 ± 0.28
*Xerocomus subtomentosus* cap	3.35 ± 0.07	12.89 ± 1.52	1.38 ± 0.06	5.90 ± 0.19
*Cantharellus cibarius*	0.77 ± 0.03	2.39 ± 0.23	0.24 ± 0.03	0.42 ± 0.04
*Agaricus bisporus* cap without peel	1.90 ± 0.09	4.80 ± 0.36	0.41 ± 0.01	2.41 ± 0.05
*Agaricus bisporus* stalk without peel	1.01 ± 0.20	3.88 ± 0.13	0.17 ± 0.04	1.4 ± 0.05
*Agaricus bisporus* cap with peel	2.04 ± 0.13	5.23 ± 0.49	0.50 ± 0.11	2.812 ± 0.06
*Agaricus bisporus* stalk with peel	1.64 ± 0.89	4.54 ± 0.17	0.26 ± 0.02	1.72 ± 0.18
*Pleurotus ostreatus*	1.44 ± 0.09	5.23 ± 0.65	0.37 ± 0.20	0.31 ± 0.01
*Suillus* spp. cap	4.78 ± 0.24	6.64 ± 0.60	1.89 ± 0.04	3.14 ± 0.05
*Suillus variegatus* cap	4.15 ± 0.35	7.44 ± 0.71	3.27 ± 0.10	2.57 ± 0.13
*Suillus bovinus* cap	4.00 ± 0.07	5.48 ± 0.42	3.02 ± 0.07	3.33 ± 0.08
*Suillus bovinus* stalk	4.05 ± 0.15	2.16 ± 0.07	2.18 ± 0.10	1.12 ± 0.03
*Macrolepiota procera* cap	2.17 ± 0.39	10.30 ± 1.50	0.918 ± 0.37	5.13 ± 0.07
*Macrolepiota procera* stalk	1.95 ± 0.30	7.51 ± 0.50	0.75 ± 0.04	2.18 ± 0.03
*Tricholoma equestre* cap	1.85 ± 0.69	4.71 ± 1.12	1.65 ± 0.19	1.44 ± 0.03
*Tricholoma equestre* stalk	0.66 ± 0.30	4.00 ± 0.50	0.65 ± 0.17	0.99 ± 0.03
*Tuber mesentericum*	0.02 ± 0.02	1.65 ± 0.10	0.06 ± 0.02	0.51 ± 0.20

The concentration of total phenolics and flavonoids was estimated spectrophotometrically. Each value represents mean ± SD, *n* ≥ 3.

**Table 2:** The content of *β*-carotene and lycopene of edible Polish mushrooms (*μ*g/g of dried mushrooms).

Mushroom	*β*-carotene (*μ*g/g of dried mushrooms)		Lycopene (*μ*g/g of dried mushrooms)	
	Methanolic extract	Aqueous extract	Methanolic extract	Aqueous extract
*Boletus edulis* white cap	0.729 ± 0.025	0.402 ± 0.077	0.076 ± 0.048	0.262 ± 0.015
*Boletus edulis* white stalk	0.467 ± 0.116	0.007 ± 0.034	0.019 ± 0.007	0.058 ± 0.049
*Boletus edulis* yellow cap	1.350 ± 0.162	0.136 ± 0.056	0.069 ± 0.006	0.103 ± 0.003
*Boletus edulis* yellow stalk	0.718 ± 0.079	0.098 ± 0.007	0.062 ± 0.017	0.087 ± 0.001
*Xerocomus badius* cap	0.752 ± 0.007	0.114 ± 0.001	0.038 ± 0.028	0.084 ± 0.007
*Xerocomus badius* stalk	2.626 ± 0.336	0.184 ± 0.025	0.461 ± 0.173	0.102 ± 0.002
*Leccinum* spp. cap	0.683 ± 0.075	0.113 ± 0.017	0.114 ± 0.024	0.086 ± 0.014
*Leccinum* spp. stalk	0.270 ± 0.049	0.098 ± 0.046	0.068 ± 0.084	0.082 ± 0.025
*Xerocomus subtomentosus* cap	3.307 ± 0.271	0.163 ± 0.009	0.379 ± 0.029	0.110 ± 0.002
*Cantharellus cibarius*	3.275 ± 0.053	0.499 ± 0.027	0.105 ± 0.044	0.124 ± 0.025
*Agaricus bisporus* cap without peel	0.511 ± 0.038	0.499 ± 0.022	0.117 ± 0.020	0.374 ± 0.023
*Agaricus bisporus* stalk without peel	0.384 ± 0.028	0.069 ± 0.026	0.011 ± 0.005	0.137 ± 0.042
*Agaricus bisporus* cap with peel	0.426 ± 0.011	0.045 ± 0.074	0.109 ± 0.015	0.047 ± 0.030
*Agaricus bisporus* stalk with peel	0.519 ± 0.029	0.107 ± 0.026	0.051 ± 0.022	0.107 ± 0.005
*Pleurotus ostreatus*	0.317 ± 0.008	0.001 ± 0.006	0.195 ± 0.005	0.009 ± 0.008
*Suillus* spp. cap	6.242 ± 0.540	0.200 ± 0.079	1.951 ± 0.153	0.127 ± 0.039
*Suillus variegatus* cap	7.730 ± 0.484	0.016 ± 0.028	1.219 ± 0.025	0.048 ± 0.035
*Suillus bovinus* cap	15.256 ± 0.785	3.382 ± 0.204	15.388 ± 0.998	3.464 ± 0.108
*Suillus bovinus* stalk	11.016 ± 0.470	0.584 ± 0.037	7.347 ± 0.644	0.420 ± 0.007
*Macrolepiota procera *cap	0.265 ± 0.019	0.192 ± 0.151	0.023 ± 0.011	0.157 ± 0.108
*Macrolepiota procera* stalk	0.319 ± 0.034	0.012 ± 0.006	0.058 ± 0.007	0.030 ± 0.012
*Tricholoma equestre* cap	18.649 ± 0.024	1.905 ± 0.268	0.001 ± 0.022	0.013 ± 0.006
*Tricholoma equestre* stalk	4.753 ± 0.271	0.439 ± 0.013	0.125 ± 0.065	0.039 ± 0.012
*Tuber mesentericum*	0.233 ± 0.053	0.098 ± 0.024	0.001 ± 0.002	0.084 ± 0.006

The content of *β*-carotene and lycopene in the extracts of dried mushrooms was estimated spectrophotometrically after extraction with the acetone: hexane mixture. Each value represents mean ± SD, *n* ≥ 3.

**Table 3:** Trolox equivalent anitoxidant capacity (*μ*mol of Trolox/g dried mushroom) and reducing power (*μ*g of gallic acid/g of dried mushroom) of edible Polish mushrooms.

Mushroom	TEAC (*μ*mol of Trolox/g dried mushroom)		Reducing power (*μ*g of gallic acid/g of dried mushroom)	
	Methanolic extract	Aqueous extract	Methanolic extract	Aqueous extract
*Boletus edulis* white cap	19.81 ± 0.35	62.30 ± 1.77	5.75 ± 0.45	13.37 ± 0.69
*Boletus edulis* white stalk	17.58 ± 1.55	46.68 ± 1.66	6.57 ± 0.46	14.60 ± 1.04
*Boletus edulis* yellow cap	19.68 ± 1.00	42.76 ± 1.66	5.18 ± 0.39	9.52 ± 0.30
*Boletus edulis* yellow stalk	19.38 ± 2.94	30.49 ± 4.38	8.09 ± 0.24	9.10 ± 1.69
*Xerocomus badius* cap	6.72 ± 0.73	40.85 ± 3.12	2.21 ± 0.32	8.14 ± 0.33
*Xerocomus badius* stalk	11.80 ± 2.53	40.34 ± 1.33	2.51 ± 0.20	8.57 ± 0.42
*Leccinum* spp. cap	9.44 ± 0.79	43.38 ± 3.39	3.80 ± 0.36	10.21 ± 0.16
*Leccinum* spp. stalk	4.62 ± 2.57	16.28 ± 1.27	1.81 ± 0.13	6.85 ± 0.35
*Xerocomus subtomentosus* cap	12.22 ± 0.40	52.49 ± 1.94	5.51 ± 0.42	13.86 ± 1.35
*Cantharellus cibarius*	0.99 ± 0.77	3.81 ± 0.17	1.80 ± 0.74	2.03 ± 0.07
*Agaricus bisporus* cap without peel	8.48 ± 1.13	13.43 ± 1.02	1.69 ± 0.30	2.09 ± 0.23
*Agaricus bisporus* stalk without peel	4.71 ± 1.81	13.19 ± 0.59	1.30 ± 0.05	3.08 ± 0.15
*Agaricus bisporus* cap with peel	9.36 ± 2.16	14.86 ± 0.51	1.85 ± 0.29	2.04 ± 0.26
*Agaricus bisporus* stalk with peel	6.16 ± 1.04	14.20 ± 0.19	1.64 ± 0.07	4.17 ± 0.26
*Pleurotus ostreatus*	4.79 ± 0.48	13.08 ± 0.37	1.47 ± 0.29	2.76 ± 0.23
*Suillus* spp. cap	19.68 ± 0.24	32.65 ± 1.99	6.00 ± 0.61	7.08 ± 0.25
*Suillus variegatus* cap	17.92 ± 1.19	23.52 ± 0.36	5.78 ± 0.50	6.31 ± 0.60
*Suillus bovinus* cap	18.25 ± 1.33	12.76 ± 0.22	4.91± 0.47	5.03 ± 0.570
*Suillus bovinus* stalk	20.54 ± 1.89	8.31 ± 0.07	4.59 ± 0.28	2.43 ± 0.30
*Macrolepiota procera *cap	8.92 ± 0.04	36.08 ± 0.37	2.25 ± 0.11	8.99 ± 0.25
*Macrolepiota procera* stalk	5.09 ± 0.89	21.83 ± 3.13	2.90 ± 0.08	9.64 ± 0.29
*Tricholoma equestre* cap	7.17 ± 1.70	11.48 ± 2.34	1.24 ± 0.04	2.29 ± 0.41
*Tricholoma equestre* stalk	1.75 ± 0.34	10.22 ± 4.11	0.59 ± 0.01	2.74 ± 0.21
*Tuber mesentericum*	0.42 ± 1.48	6.01 ± 0.36	0.13 ± 0.02	1.17 ± 0.24

Trolox equivalent antioxidant capacity and reducing power were determined spectrophotometrically. Each value represents mean ± SD, *n* ≥ 3.

**Table 4:** The correlation between the concentrations of antioxidants and TEAC/reducing power.

	*r*	
	TEAC	Reducing power
Total phenolics (aqueous extract)	0.95 ± 0.02^d^	0.92 ± 0.03^d^
Total phenolics (methanolic extract)	0.98 ± 0.01^d^	0.93 ± 0.03^d^
Flavonoids (aqueous extract)	0.68 ± 0.11^b^	0.57 ± 0.14^a^
Flavonoids (methanolic extract)	0.58 ± 0.14^d^	0.45 ± 0.17^b^
*β*-carotene (aqueous extract)	−0.27 ± 0.19	−0.09 ± 0.21
*β*-carotene (methanolic extract)	0.28 ± 0.19	0.25 ± 0.20
Lycopene (aqueous extract)	−0.16 ± 0.20	−0.23 ± 0.20
Lycopene (methanolic extract)	0.41 ± 0.17^a^	0.10 ± 0.21

The Pearson correlation coefficient between TEAC/reducing power and the concentration of antioxidants was calculated and tested with Student's *t*-test: ^a^ *P* ≤ .05, ^b^ *P* ≤ .01, ^c^ *P* ≤ .005, and ^d^ *P* ≤ .001.

## References

[1] Chang R (1996). Functional properties of edible mushrooms. *Nutrition Reviews*.

[2] Wasonga CGO, Okoth SA, Mukuria JC, Omwandho COA (2008). Mushroom polysaccharide extracts delay progression of carcinogenesis in mice. *Journal of Experimental Therapeutics and Oncology*.

[3] Li H, Lu X, Zhang S, Lu M, Liu H (2008). Anti-inflammatory activity of polysaccharide from *Pholiota nameko*. *Biochemistry*.

[4] Adams LS, Phung S, Wu X, Ki L, Chen S (2008). White button mushroom (*Agaricus bisporus*) exhibits antiproliferative and proapoptotic properties and inhibits prostate tumor growth in athymic mice. *Nutrition and Cancer*.

[5] Mori K, Kobayashi C, Tomita T, Inatomi S, Ikeda M (2008). Antiatherosclerotic effect of the edible mushrooms *Pleurotus eryngii* (Eringi), *Grifola frondosa* (Maitake), and *Hypsizygus marmoreus* (Bunashimeji) in apolipoprotein E-deficient mice. *Nutrition Research*.

[6] Sakata T (1995). A very-low-calorie conventional Japanese diet: its implications for prevention of obesity. *Obesity Research*.

[7] Fukushima M, Ohashi T, Fujiwara Y, Sonoyama K, Nakano M (2001). Cholesterol-lowering effects of maitake (*Grifola frondosa*) fiber, shiitake (*Lentinus edodes*) fiber, and enokitake (*Flammulina velutipes*) fiber in rats. *Experimental Biology and Medicine*.

[8] De Román M, Boa E, Woodward S (2006). Wild-gathered fungi for health and rural livelihoods. *Proceedings of the Nutrition Society*.

[9] Barros L, Venturini BA, Baptista P, Estevinho LM, Ferreira ICFR (2008). Chemical composition and biological properties of Portuguese wild mushrooms: a comprehensive study. *Journal of Agricultural and Food Chemistry*.

[10] Chen DI, Dou QP (2008). Tea polyphenols and their roles in cancer prevention and chemotherapy. *International Journal of Molecular Sciences*.

[11] Lambert JD, Hong J, Yang GY, Liao J, Yang CS (2005). Inhibition of carcinogenesis by polyphenols: evidence from laboratory investigations. *The American Journal of Clinical Nutrition*.

[12] Markus MA, Morris BJ (2008). Resveratrol in prevention and treatment of common clinical conditions of aging. *Clinical Interventions in Aging*.

[13] Biesalski HK (2007). Polyphenols and inflammation: basic interactions. *Current Opinion in Clinical Nutrition and Metabolic Care*.

[14] Rahman I, Biswas SK, Kirkham PA (2006). Regulation of inflammation and redox signaling by dietary polyphenols. *Biochemical Pharmacology*.

[15] Rossi L, Mazzitelli S, Arciello M, Capo CR, Rotilio G (2008). Benefits from dietary polyphenols for brain aging and Alzheimer’s disease. *Neurochemical Research*.

[16] Halpern MJ, Dahlgren AL, Laakso I, Seppänen-Laakso T, Dahlgren J, Mcanulty PA (1998). Red-wine polyphenols and inhibition of platelet aggregation: possible mechanisms, and potential use in health promotion and disease prevention. *Journal of International Medical Research*.

[17] Zern TL, Fernandez ML (2005). Cardioprotective effects of dietary polyphenols. *Journal of Nutrition*.

[18] Sies H (1992). Carotenoids and tocopherols as antioxidants and singlet oxygen quenchers. *Journal of Nutritional Science and Vitaminology*.

[19] Di Mascio P, Kaiser SP, Thomas, Devasagayam PA, Sies H (1991). Biological significance of active oxygen species: in vitro studies on singlet oxygen-induced DNA damage and on the singlet oxygen quenching ability of carotenoids, tocopherols and thiols. *Advances in Experimental Medicine and Biology*.

[20] Frei B (1994). Reactive oxygen species and antioxidant vitamins: mechanisms of action. *American Journal of Medicine*.

[21] Dahan K, Fennal M, Kumar NB (2008). Lycopene in the prevention of prostate cancer. *Journal of the Society for Integrative Oncology*.

[22] Kristal AR, Platz EA, Parnes HL, Albanes D (2004). Vitamin A, retinoids and carotenoids as chemopreventive agents for prostate cancer. *Journal of Urology*.

[23] Liu C, Russell RM (2008). Nutrition and gastric cancer risk: an update. *Nutrition Reviews*.

[24] Lotan R (1997). Retinoids and chemoprevention of aerodigestive tract cancers. *Cancer and Metastasis Reviews*.

[25] Rao AV, Rao LG (2007). Carotenoids and human health. *Pharmacological Research*.

[26] Tapiero H, Townsend DM, Tew KD (2004). The role of carotenoids in the prevention of human pathologies. *Biomedicine and Pharmacotherapy*.

[27] Riccioni G, Mancini B, Di Ilio E, Bucciarelli T, D’Orazio N (2008). Protective effect of lycopene in cardiovascular disease. *European Review for Medical and Pharmacological Sciences*.

[28] Wojewoda W (2003). *Checklist of Polish Larger Basidiomycetes*.

[29] Ławrynowicz M (1999). Tuber mesentericum an interesting species of black truffles in Poland. *Acta Mycologica*.

[30] Puttaraju NG, Venkateshaiah SU, Dharmesh SM, Urs SMN, Somasundaram R (2006). Antioxidant activity of indigenous edible mushrooms. *Journal of Agricultural and Food Chemistry*.

[31] Singleton V, Rossi J (1965). Colorimetry of total phenolics with phosphomolybdic–phosphotungstic acid reagents. *American Journal of Enology and Viticulture*.

[32] Ainsworth EA, Gillespie KM (2007). Estimation of total phenolic content and other oxidation substrates in plant tissues using Folin-Ciocalteu reagent. *Nature Protocols*.

[33] Jia Z, Tang M, Wu J (1999). The determination of flavonoid contents in mulberry and their scavenging effects on superoxide radicals. *Food Chemistry*.

[34] Barros L, Ferreira MJ, Queirós B, Ferreira ICFR, Baptista P (2007). Total phenols, ascorbic acid, *β*-carotene and lycopene in Portuguese wild edible mushrooms and their antioxidant activities. *Food Chemistry*.

[35] Nagata M, Yamashita I (1992). Simple method for simultaneous determination of chlorophyll and carotenoids in tomato fruit. *Nippon Shokuhin Kogyo Gakkaish*.

[36] Bartosz G (2003). *The Other Face of Oxygen. Free Radicals in Nature*.

[37] Re R, Pellegrini N, Proteggente A, Pannala A, Yang M, Rice-Evans C (1999). Antioxidant activity applying an improved ABTS radical cation decolorization assay. *Free Radical Biology and Medicine*.

[38] Cheung LM, Cheung PCK, Ooi VEC (2003). Antioxidant activity and total phenolics of edible mushroom extracts. *Food Chemistry*.

[39] Dubost NJ, Ou B, Beelman RB (2007). Quantification of polyphenols and ergothioneine in cultivated mushrooms and correlation to total antioxidant capacity. *Food Chemistry*.

[40] Lee JS, Park BC, Ko YUJ (2008). *Grifola frondosa* (Maitake mushroom) water extract inhibits vascular endothelial growth factor-induced angiogenesis through inhibition of reactive oxygen species and extracellular signal-regulated kinase phosphorylation. *Journal of Medicinal Food*.

[41] Sarikurkcu C, Tepe B, Yamac M (2008). Evaluation of the antioxidant activity of four edible mushrooms from the Central Anatolia, Eskisehir—Turkey: *Lactarius deterrimus, Suillus collitinus, Boletus edulis, Xerocomus chrysenteron*. *Bioresource Technology*.

[42] Watanabe T, Nakajima Y, Konishi T (2008). In vitro and in vivo anti-oxidant activity of hot water extract of Basidiomycetes-X, newly identified edible fungus. *Biological and Pharmaceutical Bulletin*.

[43] Lakshmi B, Ajith TA, Jose N, Janardhanan KK (2006). Antimutagenic activity of methanolic extract of *Ganoderma lucidum* and its effect on hepatic damage caused by benzo[a]pyrene. *Journal of Ethnopharmacology*.

[44] Wong KL, Chao HH, Chan P, Chang LIP, Liu CF (2004). Antioxidant activity of *Ganoderma lucidum* in acute ethanol-induced heart toxicity. *Phytotherapy Research*.

[45] Jayakumar T, Sakthivel M, Thomas PA, Geraldine P (2008). Pleurotus ostreatus, an oyster mushroom, decreases the oxidative stress induced by carbon tetrachloride in rat kidneys, heart and brain. *Chemico-Biological Interactions*.

[46] Kähkönen MP, Hopia AI, Vuorela HJ (1999). Antioxidant activity of plant extracts containing phenolic compounds. *Journal of Agricultural and Food Chemistry*.

[47] Ben-Amotz A, Fishler R (1998). Analysis of carotenoids with emphasis on 9-cis *β*-carotene in vegetables and fruits commonly consumed in Israel. *Food Chemistry*.

[48] Meydani M, Martin A, Ribaya-Mercado JD, Gong J, Blumberg JB, Russell RM (1994). *β*-carotene supplementation increases antioxidant capacity of plasma in older women. *Journal of Nutrition*.

[49] Pool-Zobel BL, Bub A, Müller H, Wollowski I, Rechkemmer G (1997). Consumption of vegetables reduces genetic damage in humans: first results of a human intervention trial with carotenoid-rich foods. *Carcinogenesis*.

[50] Ko SH, Choi SW, Ye SK, Cho BEL, Kim HS, Chung MH (2005). Comparison of the antioxidant activities of nine different fruits in human plasma. *Journal of Medicinal Food*.

[51] Pilaczynska-Szczesniak L, Skarpanska-Steinborn A, Deskur E, Basta P, Horoszkiewicz-Hassan M (2005). The influence of chokeberry juice supplementation on the reduction of oxidative stress resulting from an incremental rowing ergometer exercise. *International Journal of Sport Nutrition and Exercise Metabolism*.

[52] Camelini CM, Maraschin M, De Mendonça MM, Zucco C, Ferreira AG, Tavares LA (2005). Structural characterization of *β*-glucans of Agaricus brasiliensis in different stages of fruiting body maturity and their use in nutraceutical products. *Biotechnology Letters*.

[53] Dikeman CL, Bauer LL, Flickinger EA, Fahey GC (2005). Effects of stage of maturity and cooking on the chemical composition of select mushroom varieties. *Journal of Agricultural and Food Chemistry*.

[54] Barros L, Baptista P, Ferreira ICFR (2007). Effect of Lactarius piperatus fruiting body maturity stage on antioxidant activity measured by several biochemical assays. *Food and Chemical Toxicology*.

[55] Ajila CM, Prasada Rao UJS (2008). Protection against hydrogen peroxide induced oxidative damage in rat erythrocytes by *Mangifera indica L.* peel extract. *Food and Chemical Toxicology*.

[56] Ma YQ, Chen JC, Liu DH, Ye XQ (2008). Effect of ultrasonic treatment on the total phenolic and antioxidant activity of extracts from citrus peel. *Journal of Food Science*.

[57] Singh N, Rajini PS (2008). Antioxidant-mediated protective effect of potato peel extract in erythrocytes against oxidative damage. *Chemico-Biological Interactions*.

[58] Barros L, Cruz T, Baptista P, Estevinho LM, Ferreira ICFR (2008). Wild and commercial mushrooms as source of nutrients and nutraceuticals. *Food and Chemical Toxicology*.

[59] Chodorowski Z, Anand JS, Grass M (2003). Acute poisoning with Tricholoma equestre of five-year old child. *Przeglad lekarski*.

[60] Chodorowski Z, Waldman W, Sein Anand J (2002). Acute poisoning with Tricholoma equestre. *Przeglad Lekarski*.

[61] Nieminen P, Kärjä V, Mustonen AM (2009). Myo- and hepatotoxic effects of cultivated mushrooms in mice. *Food and Chemical Toxicology*.

